# Beyond intervention into daily life: A systematic review of generalisation following social communication interventions for young children with autism

**DOI:** 10.1002/aur.2264

**Published:** 2020-01-14

**Authors:** Sophie Carruthers, Andrew Pickles, Vicky Slonims, Patricia Howlin, Tony Charman

**Affiliations:** ^1^ Department of Psychology, Institute of Psychiatry, Psychology and Neuroscience King's College London London United Kingdom; ^2^ Department of Biostatistics and Health Informatics, Institute of Psychiatry, Psychology and Neuroscience King's College London London United Kingdom; ^3^ Guy's and St Thomas' NHS Foundation Trust (Evelina Children's Hospital) London United Kingdom

**Keywords:** autism, learning, generalization, intervention research, social communication, skill learning

## Abstract

Researchers have generally considered autistic individuals to have difficulties generalising learned skills across novel contexts. Successful generalisation is necessary for an intervention to have benefits in everyday life beyond the original learning environment. We conducted a systematic review of randomised controlled trials of early social communication interventions for children with autism in order to explore generalisation and its measurement. We identified nine RCTs that provided evidence of initial target learning and measured generalisation, of which eight demonstrated at least some successful generalisation across people, settings, and/or activities. The findings did not support the widely reported generalisation ‘difficulties’ associated with autism. However, generalisation was not consistent across all skills within studies, and one study found no generalisation despite evidence for initial target learning within the intervention context. In general, there are few methodologically sound social communication intervention studies exploring generalisation in autism and no consensus on how it should be measured. In particular, failure to demonstrate initial learning of target skills within the intervention setting and an absence of formal mediation analyses of the hypothesised mechanisms limit current research. We outline a framework within which measurement of generalisation can be considered for use in future trials. To maximise the effectiveness of interventions, the field needs to gain a better understanding of the nature of generalisation among autistic individuals and what additional strategies may further enhance learning. ***Autism Res** 2020, 13: 506–522*. © 2020 The Authors. *Autism Research published by International Society for Autism Research* published by Wiley Periodicals, Inc.

**Lay Summary:**

It is generally considered that autistic individuals experience difficulties applying things they have learned in one context into different settings (e.g. from school to home). This is important to consider for intervention studies. Our review does not support a complete lack of generalisation but instead suggests that after early social communication intervention, autistic children can transfer some skills to new contexts. Overall, there is limited research in this area and further work is needed.

## Introduction

Generalisation is the ability to apply learned behaviours in contexts other than the one in which it was initially acquired and may occur across different people, settings, behaviours and/or time [Stokes & Baer, 1977]. Individuals diagnosed with autism spectrum disorder (hereafter referred to as autism) are often reported as showing poor generalisation to novel settings post‐intervention [Hwang & Hughes, 2000; Lovaas, Koegel, Simmons, & Long, 1973; National Research Council, 2001; Ozonoff & Miller, 1995]. As generalisation is necessary for learned skills to be implemented in everyday life, failure to generalise would substantially limit the value of any intervention. However, there is no consensus on the extent or profile of generalisation following interventions for autistic individuals.

The process of applying what has been learned from the original context to new situations is of central importance in general development. In typical development, generalisation begins in the first few months of life and is well established by 2 years of age [Bahrick, 2002; Barnat, Klein, & Meltzoff, 1996; Morrongiello, Lasenby, & Lee, 2003]. For example, delayed imitation skills (i.e. production of words, phrases or gestures in different situations or with different people sometime after first hearing/seeing them) generalise across contexts before 12 months of age [Hayne, Boniface, & Barr, 2000], and joint engagement is seen across different play partners by 18 months [Bakeman & Adamson, 1984].

The generalisation of a newly taught behaviour across different settings, people or materials/activities is of particular importance for intervention research. A number of associative learning models have attempted to explain the mechanisms by which generalisation across contexts takes place [Atkinson & Estes, 1962; Pearce, 1987; Rescorla & Wagner, 1972]. Most of these theories assume that generalisation occurs as a function of ‘stimulus similarity’ [Byrom & Murphy, 2014]; thus, the higher number of features shared across the original and new contexts, the more readily generalisation will occur [McClelland & Rumelhart, 1985; Pearce, 1987; Rescorla & Wagner, 1972].

There are also other cognitive theories relevant to generalisation in autism [Brown & Bebko, 2012]. If generalisation is driven by the number of shared features across contexts, differences in generalisation could stem from how and where autistic individuals focus their attention, or what they consider salient in a particular context [Baron‐Cohen, 2002; Happe & Frith, 2006; Lovaas, Koegel, & Schreibman, 1979; Milton, 2017; Mottron, Dawson, Soulieres, Hubert, & Burack, 2006; Murray, Lesser, & Lawson, 2005; Plaisted, 2001], and/or how learned information is processed, organised and retrieved in memory [Baez & Ibanez, 2014; Church et al., 2015; McClelland, 2000; Miller, Odegard, & Allen, 2014; Schneider, Slaughter, Bayliss, & Dux, 2013; Williams, Goldstein, & Minshew, 2006]. These different perceptual and/or cognitive processes may mean that the features considered shared across two contexts by non‐autistic individuals may not consistently align with what autistic individuals perceive to be common elements. However, there have been few attempts to test the validity of these, or other theories of generalisation in autism and existing experimental studies are characterised by very disparate methodologies and inconsistent findings. Preliminary evidence from dot‐probe tasks [Bott, Brock, Brockdorff, Boucher, & Lamberts, 2006; Church et al., 2010; Froehlich et al., 2012; Mercado 3rd et al., 2015; Vladusich, Olu‐Lafe, Kim, Tager‐Flusberg, & Grossberg, 2010], labelling of objects and pictures [Hartley & Allen, 2014, 2015] and transfer of cognitive strategies [de Marchena, Eigsti, & Yerys, 2015] present a varied picture of the extent of any specific difficulties in generalisation associated with autism. Such studies are also limited by their use of generalisation contexts that are very similar to those of the learning environment and the fact that generalisation is assessed almost immediately after learning. Thus, they provide little information about the applicability of the findings to generalisation in daily life.

More practically relevant information on generalisation in autism may be provided by intervention trials that include measures of behaviour generalisation beyond the immediate treatment setting. Over 40 years ago, Stokes and Baer [1977] proposed that generalisation was an active process and that specific strategies should be programmed into interventions to directly target its occurrence. The authors outlined nine approaches researchers could take to support generalisation. These were later summarised by Stokes and Osnes [1989] into three principles: Exploit Current Functional Contingencies (e.g. use of reinforcement); Train Diversely (e.g. with sufficient and diverse examples); and Incorporate Functional Mediators (e.g. include common characteristics of the original learning environment and the new contexts). These principles have developed into a range of commonly used strategies, such as use of positive reinforcement, and the involvement of parents, teachers, and peers as mediators of interventions, and generalisation has increasingly become an explicit focus of many autism intervention programmes [Green & Garg, 2018; Lord et al., 2005].

Within single‐subject design intervention research, there are many examples of assessing generalisation by using structured and specific ‘probes’ (i.e. prompts) for behaviours. Several existing reviews of this literature have systematically investigated generalisation effects across a variety of intervention approaches (e.g. parent‐mediated interventions, tablet‐based interventions) [Hong et al., 2018; Hong, Neely, Gerow, & Gann, 2018; Neely et al., 2016; Schlosser & Lee, 2000]. In three such reviews, pooled effect sizes suggested target behaviours were, on average, more frequent in generalisation contexts after intervention relative to pre‐intervention and, on average, were as frequent in generalisation contexts as in the intervention contexts post‐intervention. However, there was large variation across and within individual studies and sample sizes were small [Hong, Kawaminami, et al., 2018; Hong, Neely, et al., 2018; Neely et al., 2016]. This variation within and across studies has been highlighted by several other reviews [Jung & Sainato, 2013; Kabashi & Kaczmarek, 2017; Neely, Garcia, Bankston, & Green, 2018; Shukla‐Mehta, Miller, & Callahan, 2010; van der Meer & Rispoli, 2010; Whalon, Conroy, Martinez, & Werch, 2015]. Overall, the experimental and single‐subject design literatures do not currently support a pervasive lack of generalisation in autism following intervention, but methodologies vary and findings are inconsistent. Consideration of generalisation within more focused areas of research (e.g. specific age range, type of intervention, or clinical profile) could facilitate our understanding of when generalisation does or does not occur. This could also increase awareness of factors that support the application of learning across contexts, with direct implications for enhancing intervention design.

Compared with the single‐subject design literature, there has been a less systematic focus on generalisation in the design of randomised control trials of autism interventions. This is despite growing evidence, from recent large RCTs, of the effectiveness of early interventions, particularly those targeting social communication skills [French & Kennedy, 2018; Green & Garg, 2018]. Such interventions are important, as many young children with autism struggle to communicate and interact with others, thereby restricting their opportunities to learn, develop, and make their needs and wants known. The inconsistent approach to investigating generalisation means there is no consensus on how generalisation should be measured within such trials. Randomised controlled trials (RCTs) of early interventions have, however, been more consistent in including measures of downstream functional skills (e.g. daily living skills). In particular, social communication intervention studies have highlighted a marked attenuation of intervention effect from proximal measurement to functional outcomes [Charman, 2011]. It is possible that a lack of generalisation could be an important underlying factor here.

**Figure 1:**
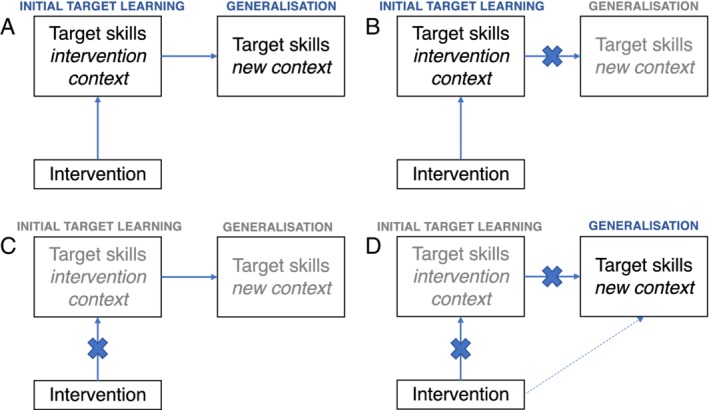
Schematics representing potential scenarios regarding initial learning and generalisation in interventions: the implied route of generalisation in interventions (**A**); problems with generalisation given successful initial target learning (**B**); poor initial learning of the target skill within the original intervention setting and therefore a knock on absence of generalisation (**C**); and presence of target skills in the generalised context despite no improvement of the target skills within the original intervention context (**D**).

Of relevance to this issue is the use of proximal (specific) vs. distal (global) outcome measures [Nordahl‐Hansen, Fletcher‐Watson, McConachie, & Kaale, 2016; Yoder, Bottema‐Beutel, Woynaroski, Chandrasekhar, & Sandbank, 2013]. Outcomes that are assessed by items or behaviours that have a high degree of overlap with the original intervention target(s) are considered *proximal*. Those items or behaviours that are related to, but broader than, the intervention target are considered as *distal* to the intervention. The context of the measure is also important. Observational measures of social communication include settings, materials, communication partners, and interaction styles. A *context‐bound* setting matches that of the original learning environment and is a test of whether the target skills were initially learned within the intervention context. Conversely, a measure of the *generalisation* of target skills is conducted in a different context to the original intervention environment [Yoder et al., 2013]. As Panel A in Figure 1 illustrates, the underlying concept of generalisation is that initial learning of the target skills within the intervention context subsequently transfers to different contexts. Therefore, to clearly evidence generalisation, at least two proximal measures are required: one of the acquisition of the target skills within the original learning context to test initial learning (i.e. context‐bound: same person, settings and materials as the intervention), and at least one measure of the target skills in a different context (i.e. generalised context: different people, settings and/or materials). What is considered a proximal measure will vary according to the narrowness of the target skills; specific individual target skills (e.g. initiations of joint attention) lend themselves to more focused measures, whereas broader targets (e.g. communication skills) may be evidenced through tools capturing a wider range of related abilities.

Within this framework, the extent of initial target learning can be presumed directly to influence the extent of subsequent generalisation. As Panel C of Figure 1 illustrates, what might be assumed to be a lack of generalisation could instead be poor initial learning of the target skill within the original intervention setting. By contrast, Panel B of the figure shows how limited generalisation would be indicated by an absence of the target skills in the new context despite improved target skills in the intervention context. Alternatively, as depicted in Panel D, the target behaviour could be present in the generalised context in the absence of evidence that the behaviour had been successfully learned within the intervention context. In other words (assuming the measures are effectively measuring the targeted constructs) the skill(s) occurring in the generalised setting may have been learned via an unintended alternative route. Within child and family interventions, while they are sometimes quite targeted on narrow proximal behaviour, they often involve a socially complex intervention that can have unexpected effects. Hence, the scenario in Panel D may be less unusual that it might at first hand seem. Without measures of initial target learning and generalisation, such relations cannot be disentangled.

### *Review Aims*

When considering the impact of any intervention, the extent of generalisation into daily life is a crucial factor. Given the importance of generalisation, and the complexity of issues surrounding its assessment, this review aimed to explore generalisation in randomised controlled trials of early interventions for autistic children that target social communication skills. We aimed systematically to explore (a) the extent to which generalisation has been measured alongside a measure of initial target learning, and (b) the evidence for generalisation following intervention.

## Methods

A systematic search was carried out in line with the guidance in the PRISMA statement [Moher, Liberati, Tetzlaff, & Altman, 2009].

### *Search Strategy and Inclusion Criteria*

Articles were identified by searching PsycInfo and PubMed. No filters were applied and there were no limitations on the year of publication. Search terms included autism or ASD or ASC or autistic and intervention* or treatment* or training or teach* (all abstract/title) and children or child or infant or toddler or pre*school or nurser* and RCT* or randomiz* or randomis* not (pharmacological or medical) (all key fields). In addition to database searches, the reference lists of relevant articles were manually searched. The first author conducted the final search on 5th August 2019.

A two‐stage screening process was implemented. The first stage identified eligible studies according to the following inclusion criteria:

- The study was a randomised controlled trial
- All participants aged below 6 years at baseline (i.e. age 0–5 years 11 months)
- All children had confirmed ASD diagnoses (diagnosis of Pervasive Developmental Disorder Not Otherwise Specified was not sufficient)
- Intervention included a focus on the acquisition of social communication skills and included measures of child outcomes
- Intervention and controls groups each had a minimum of 10 children

Studies were excluded if they involved biomedical, dietary or pharmacological interventions. Article titles and abstracts were screened by SC to identify studies fulfilling the inclusion criteria. Short‐listed articles were then separately reviewed by SC and PH. A comparison of studies identified by both authors for inclusion resulted in kappa = 0.83. Decisions about final inclusion were agreed by SC and PH.

**Figure 2:**
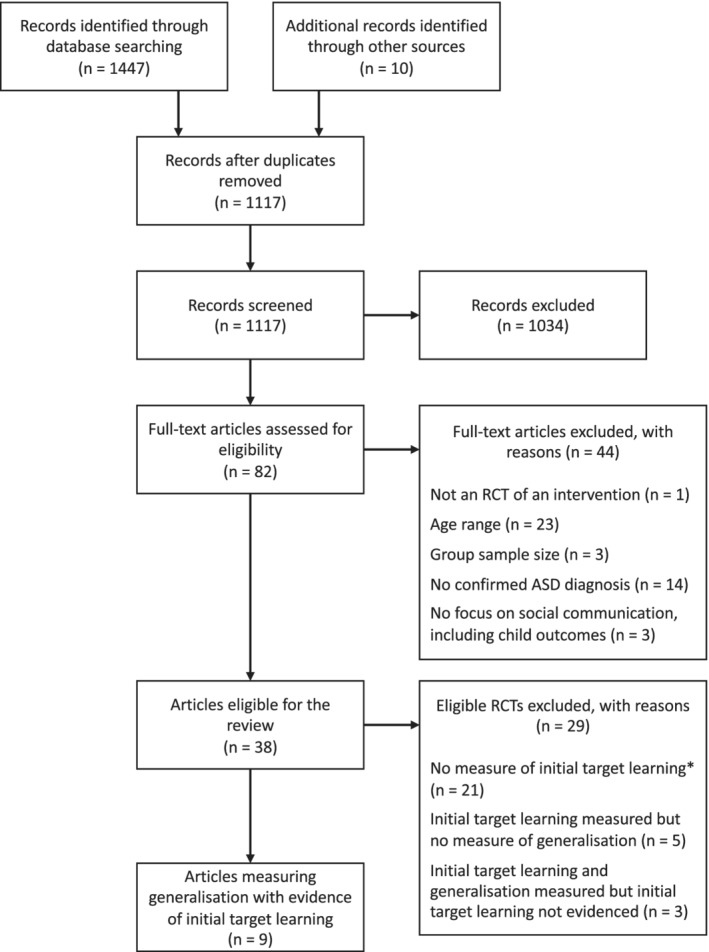
The PRISMA flow chart of study selection. *Inclusion of measures of initial target learning was assessed first and was excluded if they did not include one. These studies may or may not have included generalisation measures.

The second stage identified, out of the eligible RCTs, those that included (a) a measure of initial target learning, and (b) at least one measure of target skills in a different context for measuring generalisation. Details of these decisions are provided below. Decisions regarding eligible measurement were discussed between SC, VS, and TC and a consensus was formed on the final trials. Numbers of articles excluded at this stage, with reasons, are reported in Figure 2 and Table S2.

### *Data Extraction*

To support the second stage of screening, for each eligible RCT, the following information was extracted: intervention method(s) tested; number of participants randomised; mean participant characteristics (age, sex, cognitive level [e.g. Mullen Scales of Early Learning], autism symptom level [e.g. Autism Diagnostic Observation Schedule]); location of intervention; main provider of intervention to the child; target skills of the intervention; relevant measures and key details (location, style of task, whether blind rated); and the results associated with each measure. These details were used to inform decisions regarding which RCTs included measures of initial target learning and generalisation and to summarise results for the final studies.

### *Data Synthesis*

Each potentially eligible RCT was first categorised according to whether initial learning of the target skill within the intervention context was measured and evidenced. To assess this, the target skill and intervention context for the children were identified for each trial. If intervention was caregiver‐mediated, children were assumed to receive the intervention at home, even if the parents received guidance from a therapist in a clinic. Target skills and intervention contexts were identified through the information provided within the article. Some interventions varied in targeting micro vs. macro skills (e.g. joint attention vs. communication skills) and this was taken into account when selecting which measures were capturing initial target learning and generalisation.

To assess initial target learning, at least one measure needed to focus on the specific target skill(s) of the intervention and include the adult through whom therapy was primarily mediated (i.e. context bound). This assessment of initial learning ideally also took place in the same context as therapy, although some leeway was allowed here on account of the small number of studies identified. If a study did not include a measure of initial target learning, it was excluded and not considered further.

Generalisation measures were considered those that captured measurement of the target skills in a context that differed from the intervention context (i.e. different person, location, or materials/activity). We excluded all measures targeting downstream skills, which were often standardised tools measuring cognitive or language skills. None of the nine RCTs included specifically taught language skills during the intervention and therefore measures of vocabulary, expressive or receptive language were considered to assess global developmental progress or downstream outcomes rather than generalisation of target skills. It was decided that the Autism Diagnostic Observation Schedule (ADOS) could be used as a measure of generalisation, where applicable to the target skills, as it has been designed for this population and focuses in large part on social and communication skills. However, it may be a less precise measure of the target skills than more proximal measures. Parent‐report (or in some cases teacher‐report) were rarely considered informative for either measure of initial learning or generalisation on account of issues with precision and blinding. Standardised questionnaires often explore a range of behaviours beyond those targeted by intervention. In cases where the target skills were broader (i.e. communication), some parent or teacher report measures, or specific subscales (i.e. separate domains on the Vineland Adaptive Behaviour Scales; Sparrow, Cicchetti, & Saulnier, 2016) could provide some insight into generalisation, but were not considered optimal.

In three studies, measures of initial target learning and generalisation were included but the measure of initial target learning evidenced no effect (i.e. no evidence of initial target learning occurring), and therefore these were also excluded [Gould, 2016; Poslawsky et al., 2015; Vivanti et al., 2019]. The measures included in these studies are reported in Table S1.

There is variability within the autism intervention literature on how precisely target skills are identified and measured. Some interventions (e.g. Applied Behaviour Analysis) tend to be very specific about the particular skills being taught and tested. Developmental interventions, especially those with a focus on social interaction and communication, attempt to develop competencies (e.g. shared attention) using a range of strategies within the framework of the overall aims of the intervention. This has implications for what measures were judged relevant for initial target learning and generalisation, and these were considered on a trial‐by‐trial basis.

Decisions as to whether initial learning and generalisation were achieved were based on the presence of a significant group‐by‐time interaction effect for the relevant measure of the target skill in favour of the intervention group in comparison to the intervention as usual or low‐level intervention comparison group. An intervention effect is necessary in order to rule out change being the result of time (i.e. development) rather than learning from the intervention. The studies are grouped according to intervention type. Any use of the Stokes and Osnes [1989] principles of generalisation programming, as outlined in the introduction, are also summarised in the Results section.

## Results

### *Participant Characteristics*

The nine RCTs included 747 children aged from 2 years to 5 years 11 months. All children had a confirmed diagnosis of ASD. Table 1 provides mean values for autism severity and cognitive level for each study, where such data were reported.

**Table 1:** Summary Table of Randomised Controlled Trials Measuring generalisation

Study	Sample characteristics (age range, number randomised, gender, cognitive level, autism severity)	Intervention comparison	Target skill for children	Intervention context for children	Initial target learning assessment	Generalisation assessments
[1] Kasari et al. [2014]	Aged 2 years–5years 147 randomised 93 boys; 19 girls^a^ Mullen MA 24.9 (11.7) ADOS severity scores (*n* per module not reported): Module 1 7.6 (2.1) Module 2 6.4 (1.6) Module 3 6.9 (1.0)	Caregiver‐mediated JASPER vs. Caregiver‐only education	Joint engagement, joint attention, and play	Caregiver/home	Caregiver–child interaction^b^^,^ c Time joint engaged*	Semi‐structured researcher–child interaction (ESCS)^b^^,^ c Initiations of joint attention*
[2] Kasari, Gulsrud, Paparella, Hellemann, and Berry [2015]	Mean 31.5 months, all <36 months 86 randomised 70 boys; 16 girls Mullen DQ 68.0 (20.3) No data on level of autism symptoms	Parent‐mediated JASPER vs. Parent‐only psychoeducation	Joint engagement, joint attention, and play	Parent/home	Parent–child interaction^b^^,^ c Joint engagement* *Initiating joint attention* Functional play types* *Symbolic play types* Highest play level achieved*	Teacher–child interaction^b^ [Classroom] Joint engagement*
[3] Wong [2013]	3 years–6 years 34 randomised 29 boys; 4 girls^a^ Mullen MA 32.0 (12.9) CARS 37.8 (7.4)	Symbolic play then JA intervention vs. JA then symbolic play vs. wait‐list control	Play and joint attention	Teacher/school	Teacher–child interaction^b^ [School] Joint engagement* Joint attention responses* Joint attention initiations* *Functional play* Symbolic play* *(2 intervention groups vs. wait‐list)*	Semi‐structured researcher–child interaction (ESCS)^c^ Joint attention responses* *Joint attention initiations* Semi‐structured play with researcher (SPA)^c^ *Play level*
[4] Kaale, Smith, and Sponheim [2012]	Aged 24 months–60 months 61 randomised 48 boys; 13 girls Mullen MA 27.7 (11.5) No data on level of autism symptoms	Preschool‐based JA intervention vs. Preschool programme only	Joint attention	Teacher/school	Teacher–child interaction^b^ [Clinic at baseline, school at endpoint] Joint attention* *Joint engagement*	Mother–child play^b^ [Clinic at baseline, school at endpoint] *Joint attention* Joint engagement* Semi‐structured researcher–child interaction (ESCS)^b^ [Clinic at baseline, school at endpoint] *Joint attention*
[5] Chang, Shire, Shih, Gelfand, and Kasari [2016]	Aged 3 years–5 years 6 randomised 55 boys; 11 girls Mullen MA 35.4 (11.4) ADOS severity score 7.0 (1.3)	JASPER adapted for preschool classrooms vs. wait‐list controls	Joint attention	Teacher/school	Teacher–child interaction^b^ [School] Joint engagement* Joint attention initiations* *Initiations of behaviour requesting* Language to share: 1*/2*/3+* words Language to request: 1*/2/3+ words Play level: simple*/functional*/symbolic	Semi‐structured researcher–child interaction (ESCS)^b^ [School] *Initiations of joint attention* *Initiations of behaviour requesting* Semi‐structured play with researcher (SPA)^b^^,^ c Simple play* Functional play* *Symbolic play*
[6] Green et al. [2010]	Aged 2 years–4 years 11 months 152 randomised 124 boys; 28 girls Mullen nonverbal age 26.2 (9.8) ADOS severity score 8.0 (1.4)	Preschool autism communication therapy (PACT) vs. TAU	Communication	Parent/home	Naturalistic parent–child interaction (DCMA)^b^ [Home] Child initiations* *Shared attention*	Teacher report questionnaire (VABS)^b^ [School] *Communication subscale* Semi‐structured interaction assessment with researcher (ADOS)^b^ [Clinic] *Social affect subscale*
[7] Aldred, Green, and Adams [2004]	Aged 2;0–5;11 years 28 randomised 25 boys; 3 girls MCDI mean number of words understood 83.55 ADOS mean total score 15.9 (4.6)	‘Child's Talk’ social communication intervention vs. Routine care alone	Communication	Parent/clinic and home	Naturalistic parent–child interaction (DCMA)^b^ [Clinic] Child communicative acts* *Shared attention*	Semi‐structured interaction assessment with researcher (ADOS)^b^* [Clinic] Reciprocal Interaction* *Communication*
[8] Solomon, Van Egeren, Mahoney, Huber, and Zimmerman [2014]	Aged 2 years 8 months–5 years 11 months 128 randomised 105 boys; 23 girls Mullen visual reception 62.9 (33.9) 69.6% had autism ADOS diagnosis vs. autism spectrum disorder	The PLAY Project Home Consultation vs. Community TAU	Social reciprocity	Parent/home	Parent–child interaction (CBRS)^b^ [Home] Attention* Initiation* Parent–child interaction (FEAS)^b^ [Home] Interactional and social functioning*	Semi‐structured interaction assessment with researcher (ADOS)^b^ [Research offices] ADOS diagnostic classification*
[9] Thiemann‐Bourque, Feldmiller, Hoffman, and Johner [2018]	Aged 2;11–5;0 45 randomised 36 boys, 9 girls Mullen ELC = 49.5 (range 49–63) CARS‐2 mean 41.7, range 34.0–52.5	Peer‐mediated speech‐generating device vs. TAU with untrained peers	Communication	Peers (and iPad)/school	Interaction with familiar peer^b^ [School] Rate of total communication*	Interaction with unfamiliar peer^b^ [School] Rate of total communication* Interaction with familiar peer in novel setting or activity^b^ [School] Rate of total communication*

ADOS, Autism Diagnostic Observation Schedule; CARS, Childhood Autism Rating Scale; DCMA, Dyadic communication measure for autism; ESCS, Early Social Communication Scales; FEAS, Functional Emotional Assessment Scale; JA, Joint attention; JASPER, Joint Attention Symbolic Play Engagement and Regulation; Mullen DQ, Mullen Developmental Quotient; Mullen ELC, Mullen Early Learning Composite; Mullen MA, Mullen Mental Age; MCDI, MacArthur‐Bates Communicative Development Inventories, Words and Gestures; SPA, Structured Play Assessment; VABS, Vineland Adaptive Behaviour Scales.

*Significant group effect reported (*P* < 0.05).

a Gender data only given for participants included in final data analysis.

b Blind to group, or portion blind coded.

c Location of assessment not specified.

### *Study Characteristics*

Table 1 summarises the characteristics of the included studies. All studies provided evidence of initial target learning and measures of generalisation; these were grouped according to the type of intervention tested. Five studies^(1–5)^ were identified as using a version of the Joint Attention, Symbolic Play, Engagement and Regulation (JASPER) intervention; two^(6–7)^ used variants of the Preschool Autism Communication Trial (PACT) intervention; one^(8)^ used Play and Language for autistic youngsters (PLAY); and one^(9)^ used a peer‐mediated iPad intervention.

In relation to the Stokes and Osnes [1989] principles of generalisation programming, all nine studies incorporated common characteristics of the original learning environment and new contexts (i.e. ‘Incorporate Functional Mediators’). Five^(1–2, 6–8)^ of the studies used parents to deliver the therapies in the home setting, three^(3–5)^ used teachers in the school environment and one study^(9)^ delivered the therapy via peers within school. Furthermore, a range of cues, routines, and settings were encouraged within the interventions for the interaction‐based strategies, in line with the principle to ‘Train Diversely’ and to allow variety in the conditions of training. Finally, some of the interventions used aspects of ‘Exploiting Current Functional Contingencies’. JASPER^(1–5)^, PACT^(6–7)^, and PLAY^(8)^ interventions encourage the parents or teachers to follow the child's lead. If the child generalises targeted skills (e.g. initiates joint attention), such behaviour will be naturally reinforced with social responding and interaction from the parent (cf. study^4^). The JASPER intervention^(1–5)^ also uses other forms of positive reinforcement.

Generalisation of social communication skills was most often considered across location, person and/or style of interaction/activity. All nine studies included at least one measure of generalisation with a person other than the provider of therapy. Seven^(1, 3–8)^ included a semi‐structured or structured interaction with a researcher. These assessed generalisation not only across person, and sometimes setting, but also in style of interaction and activity. One further study^(9)^ included a measure of generalisation involving a different activity and/or setting. Three studies^(2, 6, 8)^ included measures in different locations to that of therapy. One^(4)^ study conducted assessments across different locations at baseline and endpoint; two studies^(1, 3)^ did not state where generalisation assessments took place. (See Table 1 for details of each study).

### *Generalisation*

Table 1 also summarises relevant information on the measures considered to provide evidence of initial target learning or generalisation and details pertaining to the intervention context and target skills.

#### *Joint attention symbolic play and emotion regulation*

Of the five studies using versions of JASPER, all demonstrated at least some successful generalisation. Parent or caregiver‐mediated JASPER was assessed in two studies. Kasari et al. [2014] tested parent‐mediated JASPER against a parent education intervention in low resource settings. They demonstrated greater improvement in the target skill of joint engagement on a parent–child interaction measure for those who received JASPER compared with the parent‐education group, evidencing successful initial target learning. These gains generalised to a structured assessment with a researcher, with the JASPER group demonstrating increased initiations of joint attention relative to the control group. This generalisation was maintained at 3‐month follow‐up. Initial target learning of joint engagement was also confirmed through a parent–child interaction measure in a second study of parent‐mediated JASPER [Kasari et al., 2015]. In this case, generalisation of increased joint engagement to teachers was demonstrated through a teacher–child interaction assessment in the classroom, successfully transferring across person and setting.

Three studies assessed teacher‐mediated variants of JASPER. Wong [2013] investigated the order in which the two main components of JASPER, play and joint attention, were delivered. One group received the play sessions before joint attention sessions, and the other in reverse order. A third group was in a wait‐list control group for the first 4 weeks before being randomised to receive the intervention sessions in one of the two orders. Initial target learning was evidenced in two ways. First, after the first 4 weeks, children who were in either intervention group showed greater improvements in joint engagement in the classroom, but not in play or joint attention, compared to the control group. Second, once wait‐list children went on to receive intervention, children who received the joint attention intervention first had higher rates of improvement in joint engagement and initiations of joint attention than those who received the play intervention first. Regardless of intervention order, children also improved responses to joint attention during the semi‐structured Early Social Communication Scales (ESCS) assessment with a researcher, providing some suggestions of generalisation. However, a lack of group differences in the interaction with the researcher does not rule out the possibility that the apparent generalisation effect could be a result of time. Generalisation was not found for initiations of joint attention or play skills.

Chang et al. [2016] included the measurement of several target skills. Although initial learning was evidenced with the teacher in joint engagement, initiations of joint attention, use of language to request and share, and play skills, only the improved play skills generalised to a researcher interaction. A further study evidenced improvement in joint attention with the teacher, but not joint engagement [Kaale et al., 2012]. This pattern was reversed in the generalisation context with a parent, where improvement in joint engagement, but not joint attention, was demonstrated.

In three of these studies [Kaale et al., 2012; Kasari et al., 2014; Wong, 2013], findings indicate increases in one or more of joint engagement, initiations of or responses to joint attention within the initial learning context but an increase in one of the other skills in the generalised context. As joint attention is considered to result in increased joint engagement, and joint engagement can include examples of joint attention [Kasari, Gulsrud, Wong, Kwon, & Locke, 2010], for the purpose of this review, these are considered overlapping target skills and were included as examples of generalisation.

#### *Preschool autism communication trial*

Two studies assessed versions of PACT, a parent‐mediated intervention. Aldred et al. [2004], testing an early version of what later became PACT, demonstrated that the intervention group produced a higher number of communicative acts than the control group during interaction with the parent, and generalised these gains in reciprocal interaction with a researcher as measured by the ADOS. In contrast, Green et al. [2010] showed that although children who received PACT, all of whom had core autism diagnoses, demonstrated improvement in the proportion of communication initiations made during interaction with parents at home, these gains in communication did not result in teacher‐reported improvements on the Vineland Communication subscale, or on the ADOS social affect scale with a researcher.

#### *Play and language for autistic youngsters*

Solomon et al. [2014] demonstrated that the intervention group improved in their interactional and social functioning, attention, and initiations during interactions with the parent relative to community treatment‐as‐usual (TAU). These improvements in target skills generalised to improvements in researcher‐rated assessments of social communication during the ADOS. However, the social affect and restricted and repetitive behaviour components of the ADOS were not reported separately, making this a less pure measure of generalised social communication.

#### *Peer‐mediated speech‐generating device intervention*

Thiemann‐Bourque et al. [2018] trained peers to support children with autism to use a speech‐generating device in the form of an iPad. Children receiving the intervention demonstrated significant increases in rates of communication and reciprocity towards the trained peers than those who did not have trained peers. Generalisation was evidenced with the familiar peer in a novel activity or setting and in interaction with an unfamiliar peer (although this reduced over the course of the month that it was tested).

## Discussion

This review aimed to illustrate the extent to which generalisation is currently measured and evidenced within early interventions for young children with confirmed ASD diagnoses. Of 38 potentially eligible RCTs involving some form of social communication intervention, 12 (32%) were found to include sufficient measurement of initial target learning and generalisation. Of these, nine provided evidence of successful initial target learning within the intervention context and were therefore reviewed in relation to generalisation outcomes. Eight of these studies evidenced some successful generalisation in joint attention, joint engagement, play, and/or communicative initiations. All the studies also demonstrated at least one of the Stokes and Osnes [1989] principles of programming generalisation.

### *Evidence of Generalisation*

Overall, in line with reviews of single‐subject design interventions [Hong, Kawaminami, et al., 2018; Hong, Neely, et al., 2018; Neely et al., 2016], these findings do not support the widely cited lack of generalisation associated with autism. Instead, the results suggest that young autistic children are able to generalise to contexts differing in person, setting and/or activity. However, generalisation was not found for all skills or contexts tested [Chang et al., 2016; Kaale et al., 2012; Wong, 2013] and in one study no significant generalisation effects were reported [Green et al., 2010]. Thus, it is evident that there is a continuing need to improve our understanding of the strategies that can be used to enhance and support generalisation of learning among young children with autism.

There are a few factors that may explain why the conclusions from this review appear contrary to the common belief of limited generalisation among autistic individuals. First, in accordance with our inclusion criteria, all of the included studies evidenced successful initial target learning within the intervention context. As outlined in the Introduction, without this evidence, a supposed lack of generalisation could instead be a result of a lack of initial target learning (i.e. Panel C as opposed to Panel B in Fig. 1). Sixty‐three percent (*n* = 24) of potentially eligible RCTs either did not include a measure of initial target learning or the measure used did not provide evidence of target learning having occurred. Second, the generalisation measures considered eligible here were those that were reasonably specific in their overlap (i.e. proximal) with the intervention targets. Less focused measures, such as informant questionnaires, were only considered acceptable in one study [Green et al., 2010] where a relevant subscale was reported separately. In general, informant questionnaires were not considered informative as they provided evidence of a broader range of skills beyond those focused on during the intervention (i.e. they were not sufficiently proximal). It is possible these restrictions on measurement eligibility made our evidence ‘purer’ to generalisation (as opposed to broader development) and focused on skills specifically targeted during the intervention. Finally, all nine of the studies supported generalisation by using parents, teachers or peers as ‘mediators’ of the intervention and by incorporating ‘common characteristics’ across the original learning environment and new contexts (c.f. Stokes & Baer, 1977). The use of such strategies may have further facilitated generalisation.

### *Measurement of Generalisation*

The process of identifying relevant measures of initial target learning and generalisation across the eligible RCTs revealed that out of 38 potentially eligible trials, only 12 (32%) were considered to include measures of both initial target learning and generalisation (of which nine included evidence of initial target learning). Many trials were excluded because of having no measure of initial learning within the intervention context focused on the target skills. This may reflect the common practice to include more distal outcome measures [Nordahl‐Hansen et al., 2016]. We argue that proximal measures are important for evidencing the change in target behaviour and informing on intervention mechanisms and should be used in combination with distal measures.

The most common measure of generalisation used (studies^1, 3–8^) was generalisation from parent or teacher to the researcher in semi‐structured or structured tasks (ESCS, Structured Play Assessment or ADOS). Five of these studies provided evidence of generalisation. It is important to bear in mind that semi‐structured or structured tasks with researchers, though still providing evidence for generalisation, do not fully represent many daily life scenarios. The extent of structure in any interaction observation should be considered in contrast to the more natural environments in which children typically learn and use these skills. More ecologically valid naturalistic generalisation measures would, therefore, be informative, as standardised and structured measures, by their very nature, tend to underestimate difficulties experienced in daily life [Ingersoll, 2008; Jones et al., 2011]. Nevertheless, one strength of the measures included is that most were blind‐rated observations.

### *The Path from Intervention to Generalisation*

In many intervention studies, the route from intervention to initial target learning and generalisation is implied, rather than explicitly tested. The requirement, for the present review, on having evidence of initial target learning goes some way to dealing with this issue of disentangling the different scenarios outlined in Figure 1, but ultimately mediation analyses are required to provide firm evidence of the mechanisms involved. Secondary analyses of data from two of the RCTs reviewed^(2, 6)^ included mediation analyses [Gulsrud, Hellemann, Shire, & Kasari, 2016; Pickles et al., 2015] to test the path from intervention to outcomes. In the mediation of Gulsrud et al. [2016], the hypothesised mechanism of intervention (via parental behaviour) on the initial target learning measure of parent–child interaction was confirmed. Pickles et al. [2015] further confirmed that the change in parenting behaviours (intervention strategy) not only mediates the effect on the measure of initial target learning (child initiations within parent–child interaction) but that this, in turn, mediates the effect in the generalised context of the ADOS. This mediation is instructive, even in the absence of a significant between groups' effect on the ADOS. It suggests that what change there is in the generalised context is mediated by the hypothesised mechanism of intervention effect (in this case increased parental synchrony and increased child communicative acts). Such analyses are informative for confirming the inferred route to generalisation as well as identifying active ingredients within the intervention. Together these two mediation analyses support the use of parents as ‘mediators’ of therapy in child learning and generalisation. However, the absence of mediation analyses in most studies continues to be a major shortcoming of research in this area, limiting what can be learnt from them [Green & Garg, 2018].

### *Limitations*

Given the small number of studies identified for this review, we are limited in the conclusions that can be drawn. In particular, we were not able to explore if there were differential patterns of generalisation related to the severity of autism or cognitive profiles; restrictions on the age of participants also limited analysis of possible age effects. In future research, it will be important to explore how generalisation varies across ages, intervention types and characteristics of the individuals.

Interventions can vary in the extent to which target skills are individualised for each child. Many behavioural interventions set different but related targets for each child within the framework of the intervention [e.g. Kasari, Freeman, & Paparella, 2006]. For the purpose of this review, we could only assess generalisation at the group level, but to further explore the extent and nature of generalisation among autistic children, it would be important to examine how individuals generalise the skills that were specific targets for them. Assessing generalisation at the group level may underestimate the extent of generalisation occurring.

Finally, a compromise was made during the process of identifying measures within the trials with regards to location. In order to be pragmatic and have sufficient numbers of trials to consider, we were not strict on the location of the measure of initial target learning. Of the included studies, two did not report the location of these assessments [Kasari et al., 2014, 2015], one moved the location of the assessment from clinic at baseline to school at endpoint [Kaale et al., 2012], and one had the location of the assessment in the clinic (where the parents were trained by clinicians) rather than at home where most of the child learning would have taken place [Aldred et al., 2004]. These examples are therefore likely to be less precise measures of initial target learning within the intervention context. We encourage researchers to incorporate context (i.e. location and person) in the planning of their assessments, particularly in social communication interventions where interactional context is crucial.

### *Future Research*

As indicated in the current review, good quality research into generalisation in autism is lacking. Below we highlight a number of key areas for future research.

#### *Measurement framework*

Measurement limitations hinder the interpretation of the generalisation literature and there are a number of areas where measurement needs to be improved. Summarised below is the measurement framework, as outlined in this review, that we consider necessary to measure and evidence generalisation effectively.

1. Identified intervention targets (i.e. target skills or behaviours) and reporting of specific details of context including location, main provider and style of learning (e.g. structured vs. unstructured).
2. Measure of initial learning. Proximal (i.e. focused on the same target skills as the intervention), blind‐rated measure of specific target skills conducted in a context as close to intervention as possible (i.e. with the same interventionist, in the same location, using the same materials/tasks). Sufficient detail reported to indicate any differences from the original learning context.
3. Measure of generalisation. Proximal, blind‐rated measure of the same (or at least closely overlapping) target skills in a context that differs from the intervention setting. Sufficient detail reported to determine similarities to and differences from the original learning context.

Including these steps within intervention, trials will increase the likelihood of reliably identifying, whether and to what extent, generalisation has occurred. However, where possible, RCTs should also be designed to formally measure the hypothesised mechanism of intervention effect through mediation analyses, tracing the effect of intervention to generalised outcomes via the learning of target skills (see e.g. Goldsmith, Chalder, White, Sharpe, & Pickles, 2018; Goldsmith et al., 2018). Another important issue that needs to be considered, as discussed above, is how representative of ‘real life’ generalisation the measure used actually is. The recently developed Brief Observation of Social and Communication Change [Grzadzinski et al., 2016] that affords the flexibility to be used in naturalistic adult–child interactions across contexts may be useful here [Frost, Koehn, Russell, & Ingersoll, 2019].

#### *A need for innovative research*

Existing experimental evidence on generalisation in autism mostly focuses on dot‐probe categorisation tasks, which may have limited impact when it comes to developing strategies to better support generalisation post‐intervention. Among new ideas, currently being explored is how different ways of presenting information (i.e. repeated examples of the ‘average’ of a category vs. multiple different examples of a category) can differentially support those with weaker or stronger patterns of generalisation [Church et al., 2015; Dovgopoly & Mercado 3rd., 2013]. Other strategies may include exploring different ways of structuring the learning environments. For example, preliminary work in schools has suggested that incorporating intense or ‘special’ interests in learning can be associated with improved outcomes [Wood, 2019]. Moreover, as limited or inconsistent generalisation also occurs in other neurodevelopmental conditions, such as ADHD [Abikoff, 2009; Frankel, Myatt, Cantwell, & Feinberg, 1997], it would be interesting to consider transdiagnostic intervention strategies.

Finally, the field would benefit from listening to autistic individuals and to their insights into how they learn, the strategies they use, and which situations may present greater difficulties with transferring knowledge. There is some preliminary evidence to suggest that autistic individuals may map or store knowledge differently to non‐autistic individuals or rely more on alternative methods to learn [e.g. Baez & Ibanez, 2014; Bowler, Gaigg, & Gardiner, 2008; Dawson, Mottron, & Gernsbacher, 2008]. Thus, ‘one size fits all’ teaching strategies are unlikely to be effective [Milton, 2014].

## Conclusions

Despite frequent reference in the literature to problems of generalisation in autism, early intervention trials for autistic children provide evidence that generalisation can occur post‐intervention. However, generalisation is neither consistent across skills, nor is it found in all studies. We have outlined proposals for future research, including a framework for measurement of generalisation that we hope will be useful for future studies. We believe there is real value in improving our understanding of generalisation, not only to improve the design and success of interventions but also to inform our understanding of how autistic individuals best learn in general. A more systematic and comprehensive approach to the measurement of generalisation, and how it can be enhanced, is now needed across the autism intervention literature.

## Conflict of Interest

The authors declare that they have no conflict of interest related to this work.

## Supporting information


**Appendix S1:** Supplementary materials
