# Supplementary material for: Beyond intervention into daily life: A systematic review of generalisation following social communication interventions for young children with autism
Source: Autism Res. 2020 Jan 14;13(4):506–22. doi: 10.1002/aur.2264 (PMC7187421; doi:10.1002/aur.2264)
Supplement: Supplementary file 1 — Appendix S1: Supplementary materials [file AUR-13-506-s001.docx]

**Supplementary materials**

**Beyond intervention into daily life: a systematic review of generalisation following social communication interventions for young children with autism**

**Sophie Carruthers, Andrew Pickles, Vicky Slonims, Patricia Howlin & Tony Charman**

Table S1. Summary of studies that measured initial target learning and generalisation but did not have evidence (significant effects) of initial target learning

| **Study** | **Sample characteristics**  **(age range, number randomised, gender, cognitive level, autism severity)** | **Intervention approach and comparison** | **Target skill for children** | **Intervention context for children** | **Initial target learning assessment** | **Generalisation assessments** |
| --- | --- | --- | --- | --- | --- | --- |
| **Vivanti et al 2019** | Aged 1;3-2;8  44 randomised  27 boys, 17 girls  Mullen Verbal Developmental Quotient: 57.9 (29.6)  MSEL nonverbal DQ: 74.8 (21.8)  ADOS social affect algorithm 13.6 (5.4)  ADOS RRB algorithm 4.9 (1.7) | Group ESDM in autism-specific setting  Vs.  Group ESDM in an inclusive setting | Spontaneous imitation, joint engagement, verbal and nonverbal communication | Teachers/school | Classroom observation (M-COSMIC)^‡^  [School classroom]  *Spontaneous social interaction* | Adult-child unstructured interaction (LENA)^‡¶^  *Spontaneous vocalisation*  Adult-child structured experimental interaction^‡¶^  *Spontaneous imitation* |
| **Poslawsky et al 2015** | Aged 1;4-5;1  78 randomised  67 boys; 11 girls  Mullen Composite Standard Score 73.7 (22.0)  ADOS Severity Score 7.1 (2.0) | Video feedback interaction to promote positive parenting (VIPP-AUTI) vs  Home-based nursing care | Enhancing interaction skills | Parent/home | Parent-child interaction (EAS)^‡^  [Home]  *Child responsiveness*  *Child involvement* | Examiner-child interaction (ESCS)^‡^  [Home at baseline, Hospital at endpoint]  *Initiations of joint attention*  *Responding to joint attention* |
| **Gould 2016** | Aged 33m – 54m  65 randomised  55 boys; 10 girls  Mullen Receptive Age Equivalent 19.1 (8.1)  All module 1 ADOS, no mean score provided | JASPER  vs.  Discrete Trial Training | Play skills | Mostly therapist/clinic, some parent/home | Parent-child interaction^‡^  [School, home or university clinic]  *Play type* | Semi-structured play with researcher (SPA)^‡^  [School, home or university clinic]  *Play type* |
| EAS Emotional Assessment Scales; ESCS Early Social Communication Scales; ESDM Early Start Denver Model; JASPER Joint Attention Symbolic Play Emotion Regulation; LENA Language Environment Analysis; M-COSMIC Modified Classroom Observation to Measure Intentional Communication; RRB Restricted and Repetitive Behaviours; SPA Structured Play Assessment; ^‡^ Blind to group, or portion blind coded, ^§^ Not blind, ^¶^ Location of assessment not specified | | | | | | |

Table S2. List of eligible RCTs that were not included in review with reasons

| **Study** | **Reason for exclusion** |
| --- | --- |
| Casenhiser et al. (2013) | No generalisation measure |
| Dawson et al. (2010) | No initial target learning measure |
| Drew et al. (2002) | No initial target learning measure |
| Espanola (2018) | No initial target learning measure |
| Fletcher-Watson et al. (2016) | No initial target learning measure |
| Gould (2016) | No evidence of initial target learning |
| Gulsrud et al. (2007) | No initial target learning measure |
| Ibanez et al. (2018) | No generalisation measure |
| Ingersoll (2010) | No initial target learning measure |
| Ingersoll (2012) | No initial target learning measure |
| Kasari et al. (2006) | No initial target learning measure |
| Kasari et al. (2010) | No generalisation measure |
| Kasari et al. (2008) | No initial target learning measure |
| Landa et al. (2011) | No initial target learning measure |
| Nefdt et al. (2010) | No generalisation measure |
| Poslawsky et al. (2015) | No evidence of initial target learning |
| Roberts et al. (2011) | No initial target learning measure |
| Rogers et al. (2019a) | No initial target learning measure |
| Rogers et al. (2019b) | No initial target learning measure |
| Sallows and Graupner (2005) | No initial target learning measure |
| Schreibman and Stahmer (2014) | No initial target learning measure |
| Shire et al. (2016) | No generalisation measure |
| Tonge et al. (2014) | No initial target learning measure |
| Vernon et al. (2019) | No initial target learning measure |
| Vivanti et al. (2019) | No evidence of initial target learning |
| Wetherby et al. (2014) | No initial target learning measure |
| Whitehouse et al. (2017) | No initial target learning measure |
| Yoder and Stone (2006) | No initial target learning measure |
| Yoder and Lieberman (2010) | No initial target learning measure |

**References**

Casenhiser, D. M., Shanker, S. G., & Stieben, J. (2013). Learning through interaction in children with autism: Preliminary data from asocial-communication-based intervention. Autism, 17(2), 220-241.

Dawson, G., Rogers, S., Munson, J., Smith, M., Winter, J., Greenson, J., . . . Varley, J. (2010). Randomized, controlled trial of an intervention for toddlers with autism: the Early Start Denver Model. Pediatrics, 125(1), e17-23. doi:10.1542/peds.2009-0958

Drew, A., Baird, G., Baron-Cohen, S., Cox, A., Slonims, V., Wheelwright, S., . . . Charman, T. (2002). A pilot randomised control trial of a parent training intervention for pre-school children with autism. Preliminary findings and methodological challenges. Eur Child Adolesc Psychiatry, 11(6), 266-272. doi:10.1007/s00787-002-0299-6

Espanola, E. (2018). A randomized comparison of two instructional sequences for imitation intervention for children with autism spectrum disorders. Dissertation Abstracts International: Section B: The Sciences and Engineering, 78(7-B(E)), No-Specified.

Fletcher-Watson, S., Petrou, A., Scott-Barrett, J., Dicks, P., Graham, C., O'Hare, A., . . . McConachie, H. (2016). A trial of an iPad intervention targeting social communication skills in children with autism. Autism, 20(7), 771-782. doi:10.1177/1362361315605624

Gould, H. M. (2016). Teaching to play or playing to teach: An examination of play targets and generalization in two interventions for children with autism. Dissertation Abstracts International Section A: Humanities and Social Sciences, 77(2-A(E)), No-Specified.

Gulsrud, A. C., Kasari, C., Freeman, S., & Paparella, T. (2007). Children with autism's response to novel stimuli while participating in interventions targeting joint attention or symbolic play skills. Autism, 11(6), 535-546. doi:10.1177/1362361307083255

Ibanez, L. V., Kobak, K., Swanson, A., Wallace, L., Warren, Z., & Stone, W. L. (2018). Enhancing interactions during daily routines: A randomized controlled trial of a web-based tutorial for parents of young children with ASD. Autism Research, 11(4), 667-678. doi:http://dx.doi.org/10.1002/aur.1919

Ingersoll, B. (2010). Pilot randomized controlled trial of Reciprocal Imitation Training for teaching elicited and spontaneous imitation to children with autism. J Autism Dev Disord, 40(9), 1154-1160. doi:10.1007/s10803-010-0966-2

Ingersoll, B. (2012). Brief report: effect of a focused imitation intervention on social functioning in children with autism. J Autism Dev Disord, 42(8), 1768-1773. doi:10.1007/s10803-011-1423-6

Kasari, C., Freeman, S., & Paparella, T. (2006). Joint attention and symbolic play in young children with autism: a randomized controlled intervention study. J Child Psychol Psychiatry, 47(6), 611-620. doi:10.1111/j.1469-7610.2005.01567.x

Kasari, C., Gulsrud, A. C., Wong, C., Kwon, S., & Locke, J. (2010). Randomized controlled caregiver mediated joint engagement intervention for toddlers with autism. J Autism Dev Disord, 40(9), 1045-1056. doi:10.1007/s10803-010-0955-5

Kasari, C., Paparella, T., Freeman, S., & Jahromi, L. B. (2008). Language outcome in autism: randomized comparison of joint attention and play interventions. J Consult Clin Psychol, 76(1), 125-137. doi:10.1037/0022-006x.76.1.125

Landa, R. J., Holman, K. C., O'Neill, A. H., & Stuart, E. A. (2011). Intervention targeting development of socially synchronous engagement in toddlers with autism spectrum disorder: a randomized controlled trial. J Child Psychol Psychiatry, 52(1), 13-21. doi:10.1111/j.1469-7610.2010.02288.x

Nefdt, N., Koegel, R., Singer, G., & Gerber, M. (2010). The use of a self-directed learning program to provide introductory training in pivotal response treatment to parents of children with autism. Journal of Positive Behavior Interventions, 12(1), 23-32. doi:http://dx.doi.org/10.1177/1098300709334796

Poslawsky, I. E., Naber, F. B., Bakermans-Kranenburg, M. J., van Daalen, E., van Engeland, H., & van, I. M. H. (2015). Video-feedback Intervention to promote Positive Parenting adapted to Autism (VIPP-AUTI): A randomized controlled trial. Autism, 19(5), 588-603. doi:10.1177/1362361314537124

Roberts, J., Williams, K., Carter, M., Evans, D., Parmenter, T., Silove, N., . . . Warren, A. (2011). A randomised controlled trial of two early intervention programs for young children with autism: Centre-based with parent program and home-based. Research in Autism Spectrum Disorders, 5(4), 1553-1566. doi:http://dx.doi.org/10.1016/j.rasd.2011.03.001

Rogers, S. J., Estes, A., Lord, C., Munson, J., Rocha, M., Winter, J., . . . Talbott, M. (2019a). A Multisite Randomized Controlled Two-Phase Trial of the Early Start Denver Model Compared to Treatment as Usual. J Am Acad Child Adolesc Psychiatry. doi:10.1016/j.jaac.2019.01.004

Rogers, S. J., Estes, A., Vismara, L., Munson, J., Zierhut, C., Greenson, J., . . . Talbott, M. (2019b). Enhancing low-intensity coaching in parent implemented Early Start Denver Model intervention for early autism: A randomized comparison treatment trial. J Autism Dev Disord, 49(2), 632-646. doi:http://dx.doi.org/10.1007/s10803-018-3740-5

Sallows, G. O., & Graupner, T. D. (2005). Intensive behavioral treatment for children with autism: four-year outcome and predictors. Am J Ment Retard, 110(6), 417-438. doi:10.1352/0895-8017(2005)110[417:Ibtfcw]2.0.Co;2

Schreibman, L., & Stahmer, A. C. (2014). A randomized trial comparison of the effects of verbal and pictorial naturalistic communication strategies on spoken language for young children with autism. J Autism Dev Disord, 44(5), 1244-1251. doi:10.1007/s10803-013-1972-y

Shire, S. Y., Gulsrud, A., & Kasari, C. (2016). Increasing Responsive Parent-Child Interactions and Joint Engagement: Comparing the Influence of Parent-Mediated Intervention and Parent Psychoeducation. J Autism Dev Disord, 46(5), 1737-1747. doi:10.1007/s10803-016-2702-z

Tonge, B., Brereton, A., Kiomall, M., Mackinnon, A., & Rinehart, N. J. (2014). A randomised group comparison controlled trial of 'preschoolers with autism': a parent education and skills training intervention for young children with autistic disorder. Autism, 18(2), 166-177. doi:10.1177/1362361312458186

Vernon, T. W., Holden, A. N., Barrett, A. C., Bradshaw, J., Ko, J. A., McGarry, E. S., . . . German, T. C. (2019). A Pilot Randomized Clinical Trial of an Enhanced Pivotal Response Treatment Approach for Young Children with Autism: The PRISM Model. J Autism Dev Disord, 49(6), 2358-2373. doi:10.1007/s10803-019-03909-1

Vivanti, G., Dissanayake, C., Duncan, E., Feary, J., Capes, K., Upson, S., . . . Hudry, K. (2019). Outcomes of children receiving Group-Early Start Denver Model in an inclusive versus autism-specific setting: A pilot randomized controlled trial. Autism, 23(5), 1165-1175. doi:10.1177/1362361318801341

Wetherby, A. M., Guthrie, W., Woods, J., Schatschneider, C., Holland, R. D., Morgan, L., & Lord, C. (2014). Parent-Implemented Social Intervention for Toddlers With Autism: An RCT. Pediatrics, 134(6), 1084. doi:10.1542/peds.2014-0757

Whitehouse, A. J. O., Granich, J., Alvares, G., Busacca, M., Cooper, M. N., Dass, A., . . . Anderson, A. (2017). A randomised controlled trial of an iPad-based application to complement early behavioural intervention in Autism Spectrum Disorder. Journal of Child Psychology and Psychiatry, 58(9), 1042-1052. doi:http://dx.doi.org/10.1111/jcpp.12752

Yoder, P., & Stone, W. L. (2006). Randomized comparison of two communication interventions for preschoolers with autism spectrum disorders. J Consult Clin Psychol, 74(3), 426-435. doi:10.1037/0022-006x.74.3.426

Yoder, P. J., & Lieberman, R. G. (2010). Brief Report: Randomized test of the efficacy of picture exchange communication system on highly generalized picture exchanges in children with ASD. J Autism Dev Disord, 40(5), 629-632. doi:10.1007/s10803-009-0897-y
